# Introduction to the Topic of the Special Issue “Study on Synthesis and Properties of Metal-Containing Matrix Polymer Composites” from the Guest Editor

**DOI:** 10.3390/ma16052037

**Published:** 2023-03-01

**Authors:** Oleg V. Mikhailov

**Affiliations:** Department of Analytical Chemistry, Certification and Quality Management, Kazan National Research Technological University, K. Marx Street 68, 420015 Kazan, Russia; olegmkhlv@gmail.com

As is known, one of the key objects of modern Materials Science is composite materials, or simply composites, which are used in various fields of science and technology—from the food industry to aviation, from medicine to the construction of various buildings, from agriculture to radio electronics. According to the definition adopted in Materials Science, in the general case, a composite is a poly-component system of natural or anthropogenic origin, consisting of at least two or more components with significantly different physicochemical characteristics, which, when combined with each other, lead to the appearance of some new properties that are different from those inherent in the initial components of this system. It should be specially noted in this connection that not all properties of the system are included within the definition, but only those which are not the result of the simple addition or superposition of the properties of the system’s constituent components. Each of these components can be classified into one of two categories, namely, matrix/matrices and filler/fillers, the first of which perform the function of a binder, while the latter act as a so-called binder reinforcement (strengthening the structure of the material as a result of the formation of various bonds (chemical, intermolecular, etc.) with the matrix/matrices). A special case of composites is the so-called “hybrid materials”, which are materials obtained through the interaction of chemically different constituents (components), most often organic and inorganic, forming a certain crystalline or spatial structure that differs from the structures of the original reagents (but, as a rule, retains certain motifs and functions of the original structures to one degree or another). In a number of cases, a mixture of spatially distributed phases is also considered a hybrid material (for example, if nanoparticles or nanofibers are in a polymer matrix), but it is more correct to classify as hybrid materials only those composites with a fairly obvious chemical interaction between their components. To this definition, many supramolecular compounds, including metal complexes, also correspond, but they are usually considered as a separate class of materials. Occasionally, nanoparticles with a chemically modified surface are also referred to as hybrid materials. In this case, it is usually assumed “by default” that all the components that make up the composite are in a solid state of aggregation (which can be both crystalline and amorphous), although, among experts in the field of Materials Science, there is an opinion that the matrix may also be a liquid crystal substance.

The possibilities in the composition of composites are, in principle, inexhaustible, since according to the above definition, there are no restrictions on either the range of matrices or the range of fillers. This circumstance, of course, introduces certain difficulties into their systematics. Within the framework of the most common variants—that is, by the nature of the matrix—three main categories of composites can be distinguished: polymer, metal, and ceramic, the matrices in which are, respectively, an array of any polymer or high-molecular compound, an individual metal or an alloy of two or more metals, and various types of ceramics, porcelain, faience, fireclay, majolica, etc. This classification of composites is the main one, but not the only one. Another important option is the division of composites according to the nature and structure of the filler, in the framework of which, each composite can be attributed to one of four classes: fibrous (reinforced with fibers or whiskers), layered (reinforced with films, plates, or layered fillers), dispersed-hardened (reinforced with dispersed particles of any substances), and nanocomposites (reinforced with nanoparticles). Systematics is also possible according to which of the two basic classes of chemical compounds—inorganic or organic—are included in the composition of matrices and fillers; that is, systematics according to the areas of their application in anthropogenic activities, etc. So, in the case of hybrid materials, if the “basis” of the material is organic (polymeric and other structures), then such materials are called inorganic-organic; otherwise, on the contrary, they are organic-inorganic (metal-complex frame structures, modified materials based on clays, zeolites, etc.).

Among all composites, the most diverse and widespread are undoubtedly polymer composites, which, in turn, are subdivided into six classes with a certain degree of conventionality: fiberglass (the filler of which is glass fiber), carbon fiber reinforced plastics (the filler of which are carbon fibers), boron plastics (filler—boron fibers, embedded in a thermosetting polymer matrix), organoplastics (filler—organic, synthetic, and/or natural fibers), textolites (layered plastics containing fabrics from various fibers are the filler), and polymers filled with powders of various solid (usually inorganic) substances. In addition, although such a classification of composites, in our opinion, is very “amorphous” and not clearly structured, nevertheless, it quite clearly follows that the set of composites named in it is the largest, both in terms of number and variety of materials related to it. The first in this list, fiberglass, are polymer composite materials reinforced with glass fibers, which are formed from molten inorganic glass. As the matrix, thermosetting synthetic resins (e.g., phenolic, epoxy, polyester) or thermoplastic polymers (e.g., polyamides, polyethylene, polystyrene) are used most often. These materials have sufficiently high strength, low thermal conductivity, high electrical insulation characteristics, as well as so interesting a property as transparency with respect to radio wave radiation. Composites of the second category within this classification—carbon fiber reinforced plastics as a filler contain various carbon fibers—are obtained, as a rule, from synthetic and natural fibers based on cellulose, acrylonitrile copolymers, petroleum, and coal tar pitches. It should be noticed in this connection that, for obtaining such carbon fibers, characterized by a high content of carbon (up to 99.5 mass. %), the above substances are subjected to a three-stage heat treatment, including the process of their oxidation at a temperature of 200–220 °C, carbonization at a temperature of 1000–1500 °C, and graphitization at temperature 1800–3000 °C. For obtaining carbon fiber reinforced plastics, the same matrices are used as for fiberglass plastics, namely, thermosetting and thermoplastic polymers. The main advantages of these over fiberglass are their lower density and higher modulus of elasticity; they, unlike fiberglass, are good conductors of electric current. In fairness, however, we have to note that all these polymer composites are painted black (which is understandable, since the fibers containing them are composed of graphite), and this somewhat limits their scope. Polymer composites of the third category are very similar to carbon fiber plastics—boron plastics containing boron fibers as a filler embedded in a thermosetting polymer matrix; the fibers can be both in the form of threads and in the form of bundles braided with an auxiliary glass thread or tape, in which boron threads are intertwined with other threads. Such fibers have greater hardness than carbon fibers, as a result of which boron plastics also have higher mechanical properties (in particular, boron fibers have the highest compressive strength compared to fibers from other materials and greater resistance to aggressive agents). However, the cost of boron fibers is significant, which is associated with the specific features of the technology for their production (in which boron is deposited from its trichloride onto a tungsten substrate, the cost of which can reach up to 30% of the cost of the fiber itself). Their other drawback is their very high brittleness, which makes their processing difficult and imposes restrictions on the shape of those products made from their substance. These composites are mainly used in aviation and space technology in parts subjected to long-term loads in aggressive environments. The next category of polymer composites in the order of enumeration are organoplastics; these are polymer composites in which organic, synthetic and, less commonly, natural and artificial fibers in the form of bundles, threads, fabrics, paper, and so on, serve as fillers. In these objects, the role of the matrix is played, as a rule, by thermosetting epoxy, polyester, and phenolic resins, as well as polyimides, although, in principle, organoplastics in which the matrix is thermoplastic polymers—polyethylene, polyvinyl chloride, or polyurethane—are also possible. Such composites are lighter than both fiberglass and carbon fiber and boron plastics; they have relatively high tensile strength, high impact, and dynamic resistance, but, at the same time, low compressive and flexural strength. Already a small content of filler in composites of this type leads to the appearance of qualitatively new mechanical properties of the material. The properties of the composite can also be widely varied by changing the orientation, size, and concentration of the fibers. In addition, fiber reinforcement contributes to the appearance of anisotropy in the physicochemical characteristics of the composite, and by adding conductor fibers, it is possible to impart electrical conductivity to the material along a given direction. An important role in the formation of the mechanical characteristics of organoplasty is played by the orientation of the filler macromolecules relative to each other. The macromolecules of rigid chain polymers such as polyparaphenyl terephthalamide are generally oriented in the direction of the web axis and therefore have high tensile strength along the fibers. Bulletproof body armor is made from materials reinforced with this polymer. Moreover, textolites, which are layered plastics reinforced with fabrics from various fibers, can be included among organoplastics. However, they were singled out in a separate category, since the matrix here is usually phenol–formaldehyde resin, which, unlike traditional polymeric materials, has increased brittleness. Incidentally, textolites are perhaps the oldest of all polymer composites, because the first technology for their production dates back to the early 1920s; fabrics were impregnated with resin then pressed at elevated temperature, obtaining textolite plates. It should be noted, however, that at present, the binders in textolites are a wide range of both thermosetting and thermoplastic polymers; sometimes, even inorganic binders based on silicates and phosphates are used. As a filler, fabrics from a wide variety of fibers are used—cotton, synthetic, glass, carbon, asbestos, basalt, and so on. Accordingly, the properties and application of textolites are diverse. The last category from the above list is represented by composites, which are polymers filled with powders of various substances. More than 10,000 grades of filled polymers are now known; the simplest and most ancient of them is bakelite, proposed back in 1916, the matrix of which is phenol–formaldehyde resin, and the filler is wood flour.

Among polymer composites, a specific category of physicochemical systems called “Metal-Containing Matrix Polymer Composites” stands out, which are the objects of study in the articles related to this Special Issue. An officially accepted definition of the term “Metal-Containing Matrix Polymer Composites” by the IUPAC has not yet been developed; however, it seems to us that such a definition can be planned. In our opinion, in the general case, by them we should understand all those polymer composites which include any substances containing chemical elements/metals (i.e., those chemical elements for which, in simple substances formed by them, multicenter metallic bonds are realized, positively charged metal ions are located at the nodes of the crystal lattice, and electrons move randomly between them, like gas molecules, holding positive ions together). As a rule, polyethylene, polypropylene, and polyamide fibers are used as polymer matrices since they are characterized by low cost, high stabilizing properties, and ease of heat treatment, although the list of polymers suitable for creating a matrix is much wider.

In this connection, it is possible to divide metal-containing polymer composites into three groups based on the location of the metal-containing compounds in the composite. The composites included in the first category contain particles of elemental metals and/or metal-containing substances only in the composition of the filler; the composites of the second category, only in the composition of the polymer matrix; while, for the composites of the third category, both in the composition of the polymer matrix and in the composition of the filler. In the last two cases, the polymer matrix is the so-called “metal-coordinated polymer”, in which metal elements are part of the macromolecules that make up its structure. The main ways to obtain such polymers are the polymerization of the corresponding metal-coordinated monomers and the immobilization of low-molecular-weight-metal-containing compounds in a polymer matrix. In particular, 3D-metal-coordinated polyurethanes have a complex of enhanced physical and mechanical properties and high electrical conductivity compared to traditional polyurethanes, which allows them to be used as structural polymeric materials for special purposes, both individually and as part of polymer composites.

A special place among metal-containing polymer composites is occupied by those in which the filler is composed of nanoparticles of elemental metals or metal-containing chemical compounds (the so-called “polymer nanocomposites”). The urgency of the problem associated with the synthesis, properties, and formation of such physicochemical systems is largely associated with the prospects of creating on their basis a wide variety of optical devices, in particular, miniature switches, sensors, modulators, components of the so-called “random” lasers, and devices for three-dimensional optical recording of information. The use of polymer nanocomposites is by no means limited to this; their prospects in solving the problems of ensuring electromagnetic compatibility, noise protection, radio masking, and the protection of biological objects from the harmful effects of ultrahigh-frequency radiation have been demonstrated. There is reason to believe that the use of such composite materials in microwave equipment as distributed nonlinear elements (filling waveguide paths and resonators, thin-film coating, electromagnetic screens, etc.) will make it possible to develop a number of new devices for converting electromagnetic signals and elements of active stealth technologies. In addition to these wide possibilities of practical application, it should also be noted that, unlike materials containing nanoparticles in inorganic carriers, nanocomposites based on organic polymers have the ability to be molded, which makes it easy to manufacture parts of any given shape based on these composites. It is also important that the nanoparticles contained in such polymer composites are, as it were, “cemented” in the array of the polymer matrix and, therefore, are very stable.

It seems to us that at this point in time, the following problems can be identified, which, in one way or another, are related to polymer metal-containing composites:

*Creation of scientific foundations that allow the prediction of various characteristics of polymeric metal-containing composites, determined by the nature of matrices and fillers (mechanical, physical, chemical) both at the macro, micro, and nano levels of organization of these objects*. So far, we have to state with regret that the creation of new polymeric metal-containing composites, as a rule, is carried out in a purely empirical way, reminiscent of the so-called “trial and error method”. In this connection, it becomes important, in particular, to predict the structural and geometric parameters of metal-containing composites, which determine those of their physicochemical properties that are somehow related to their practical use. This is especially true for metal-containing polymer nanocomposites, which have a number of specific chemical and physical properties that are of interest from both fundamental and applied points of view.

*Identification of regularities connected with the processes of forming polymeric metal-containing composites.* This problem is mainly of an academic nature, but nevertheless requires very careful consideration since the specifics of the processes of formation of composites, in one way or another, affect their physicochemical characteristics. These processes, as a rule, are quite complex (and sometimes difficult to predict in their results even at the modern level) since their specificity is influenced not only by the nature and state of the aggregation of the starting materials for obtaining compositions, but also by the size of their particles, geometric shape, and surface topology (and this applies to both the matrix and the filler). Due to the rapid development of nanoscience and nanotechnology, it was possible to achieve some success in understanding these patterns; however, since the number of polymer composites grows from year to year, the above problems are permanent and remain relevant. Despite the fact that at this point in time, there are a number of experimental and theoretical scientific works that consider methods for obtaining and studying the main physicochemical parameters of nanocomposites, their properties are still relatively little studied; in addition, the number of metals and their compounds that can be used as a filler is significantly limited.

*Synthesis of new polymer metal-containing composites with predetermined characteristics.* This problem is currently becoming very important due to the obvious practical solution; time and life require the emergence of new composites with improved physicochemical and/or physicomechanical parameters. Its solution, however, is directly related to the solution of the first two indicated problems, since the implementation of the processes for obtaining such composites requires not only information about what happens at one stage or another of their production, but also the possibility of purposefully controlling the process during any such stage.

*Optimization and improvement of existing methods for obtaining already known polymeric metal-containing composites.* As is known, one of the most important tasks of chemistry and Materials Science, along with the development of new methods for obtaining various substances, is the improvement of existing methods. This problem is also relevant in the case of obtaining polymer metal-containing composites, including those that are the objects of study in the articles of this Special Issue and, first of all, polymer nanocomposites.

*Improvement of systematics and chemical nomenclature of polymeric metal-containing composites.* As is easy to see, the existing systematics of composites in the broad sense of the word is based on a functional branch principle rather than a physical–chemical one; for metal-containing polymer composites, systematics can be used, which will be based on the chemical nature of the matrix and filler. On the other hand, it is necessary to somehow solve the problem associated with the compilation of the names of these composites within the framework of the modern IUPAC systematic nomenclature. This is necessary if only because it is difficult to talk about something that does not have a well-defined proper name.

Taking all of this into account, it seems to us that the number of works devoted to these interesting and important objectives of modern materials science should be significantly larger compared to what is happening at the present time. This Special Issue of *Materials* is designed, at least to some extent, to contribute to the development of the scientific direction of Materials Science associated with these objectives.

